# Healing Effects of Human Amniotic Membrane and Burned Wool on the Second-degree Burn in Rats

**DOI:** 10.31661/gmj.v9i0.1759

**Published:** 2020-12-18

**Authors:** Nader Tanideh, Farideh Keshavarzi, Aida Hemat Zadeh, Sajad Daneshi, Omid Koohi-Hosseinabadi, Maral Mokhtari, Anahita Sedighi, Seyedeh-Leili Asadi-Yousefabad

**Affiliations:** ^1^Stem Cells Technology Research Center, Shiraz University of Medical Sciences, Shiraz, Iran; ^2^Department of Pharmacology, School of Medicine, Shiraz University of Medical Sciences, Shiraz, Iran; ^3^Student Research Committee, Shiraz University of Medical Sciences, Shiraz, Iran; ^4^School of Veterinary Medicine, Shiraz University, Shiraz, Iran; ^5^Central laboratory, Shiraz University of Medical Sciences, Shiraz, Iran; ^6^Department of Pathology, School of Medicine, Shiraz University of Medical Sciences, Shiraz, Iran; ^7^Student Research Committee, Shahid Sadoughi University of Medical Sciences, Yazd, Iran

**Keywords:** Wound Healing, Rat, Burn, Pathology

## Abstract

**Background:**

This study aimed to compare sheep burnt wool and human amniotic membrane (AM) on second-degree burn wound healing in rats.

**Materials and Methods:**

Seventy-two adult male rats of Sprague Dawley underwent general anesthesia, and a deep second-degree burn was created on their skin by a hot iron plate. Afterward, human AM, silver sulfadiazine ointment (SSD), and sheep burned wool were used on wound area for burn treatment. On days 7, 14, and 21 of the experiment, the rats were sacrificed, and histopathological assessments were done.

**Results:**

Human AM, in comparison with other groups, significantly (P<0.05) showed better improvement in all pathologic variables. Burned wool showed significant improvement compared to the control group on day 7 in the angiogenesis, on day 14 in granulation tissue formation and epithelial formation, and on day 21 in new epithelial formation (P<0.05). Burned wool compared with SSD ointment in granulation tissue formation improved significantly (P<0.05) on days 7 and 14. Also, SSD ointment in comparison with the control group significantly improved (P<0.05) granulation tissue formation and macrophage on day 7.

**Conclusion:**

Human AM has a significant effect on the treatment of second-degree burn. Burned wool has a better effect on wound healing than SSD ointment and negative control group without treatment in terms of granulation tissue and epithelium formation.

## Introduction

The burn is one of the most destructive damages in the world associated with inflammation, tissue damage, infection, mortality, and disability [1]. Physical scars and psychological effects resulting from burn injury as an emergency medicine are common in developed and developing countries [2]. It seems that the government must hold educational programs about the fire to decline its emotional complications and awful event [3]. Similar physiology and tissue organization in humans and rats make it possible that rat can be applied as a burn model to reveal human tissues’ reactions under specific conditions [4]. Wound coverage using artificial, biological, and the mixed coating is very important [5]. Without it, an increase in the number of deaths, bacterial growth, and other complications will be observed [6]. Some herbal plants useful for burn wound healing have been described in China and India. Iranian traditional medicines have been scientifically studied [7,8]. Topical antimicrobial therapy is necessary for burn wounds healing. Silver sulfadiazine (SSD) ointment is used to control infection in second-degree burns. However, its side effects, including hyperosmolarity, allergic reactions, transient leukopenia, methemoglobinemia, and hemolysis were reported [9,10]. Silver may have cytotoxic effects on the fibroblast and keratinocyte, so it can inhibit wound epithelialization and delay wound healing [11]. The Alpha ointment is produced from the *Lawsonia inermis*. It has very few side effects and is more effective in infection control and wound healing [9]. Sheep burned wool is traditionally used in burn wound healing. The wool fiber has keratins and keratin-associated proteins (KAPs). An important part of the physical properties of wool is associated with KAPs [12]. The keratin-based coating may potentially improve wound healing results, recover the tissue, and avoid the need for surgery [13,14]. Keratin proteins have essential roles for wound healing; hence, controlled keratin gene (KRT) expression increased cell growth, differentiation, and migration [15]. Hair keratin-derived biomaterials are proposed as a new, simple and less costly method for therapy. Wool fibers can keep air inside [16]. The human amniotic membrane (AM) is an attractive method of grafting for wounds. AM has specific properties, such as bacteriostatic and anti-inflammatory effects, wound protection, decreased scarring, and pain reduction. Also, AM is merely available and inexpensive compared to other bioengineered skin substitutes. AM has many growth factors, cytokines, and signaling molecules that play significant roles in fetal development and gestation [17]. These molecules are also important in tissue reconstruction and wound healing [14]. A previous study showed that AM improved epithelialization [18]. Also, an increase in the success rate of grafts in the wounds and a decrease of scar formation were reported following wound dressing using AM [19,20]. The treatment of burn injuries is often long and expensive for patients, so inexpensive materials and therapeutic methods with the best effect should be used [11]. This study aimed to determine the healing effects of sheep burned wool and human AM on second-degree burn wound in rats.

## Materials and Methods

###  Experimental Animals

Seventy-two adult Sprague Dawley **(**220±20 g and 10-12 week) male rats were obtained from the Laboratory Animal Center of Shiraz University of Medical Sciences. They were housed in controlled environmental conditions (temperature 22±2ºC, humidity 55±5%, 12h light /12h dark cycle). The animals were fed with standard diet and water, *ad libitum*. Two weeks before the study, they were kept in separate cages (4 rats in each cage). This study was approved by the Ethics Committee of the Shiraz University of Medical Science (95-01-01-11592). The required wool was provided by wool spinning centers. In the beginning, the wool was washed well, and after drying, it was heated at 180^°C^ oven for 30 minutes. The burned wool in powder form was stored in a clean and sterile container. The AM was freshly prepared from the elective cesarean section in aseptic surgery condition from Hafez hospital in Shiraz city and transferred to the Laboratory Animal Center in a container containing normal saline in sterile conditions.

###  Burn Wound Model

Rats were anesthetized with ketamine 10% (100 mg/kg, Alfasan Co., Netherlands) and xylazine 2% (8 mg/kg, Alfasan Co., Netherlands). The back of the rats was shaved and disinfected. An iron plate (size: 0.5×0.5cm^2^) was exposed to flame for 5 minutes; then, it was put on the back of the rats for three seconds to induce deep second-degree burn. The wound site was washed with sterile normal saline.

###  Experimental Groups

 The animals were randomly divided into four equal groups (n=18) as fallow:

 Group I (negative control): Without any treatment.

 Group II (positive control): The surface of the wound was covered with SSD ointment daily.

 Group III: Human AM was placed on the wound and then fixed with suture on burn wound

 Group IV: Burned wool powder was placed on the wound daily so that it completely covered the surface of the wound.

###  Histopathological Analysis

Six rats from each group were randomly selected and sacrificed on days 7, 14, and 21 after induction of burn with ether inhalation for pathological examination. Tissue biopsy was taken and stored in a container containing 10% formalin and transferred to the pathology laboratory. Samples were processed, and after preparing paraffin blocks, sections with a thickness of 5 μm with a microtome were created and stained with hematoxylin and eosin (H&E). Then, the slides were evaluated in terms of re-epithelialization, granulation tissue formation, inflammatory response, macrophages, and angiogenesis [9].

###  Statistical Analysis

 Statistical data were analyzed with SPSS software (Version 21, IBM Corp, Armonk, NY). Mann-Whitney U test was used to compare the groups with the control group and different groups with each other. A P<0.05 was considered significant.

## Results

**Figure 1:**
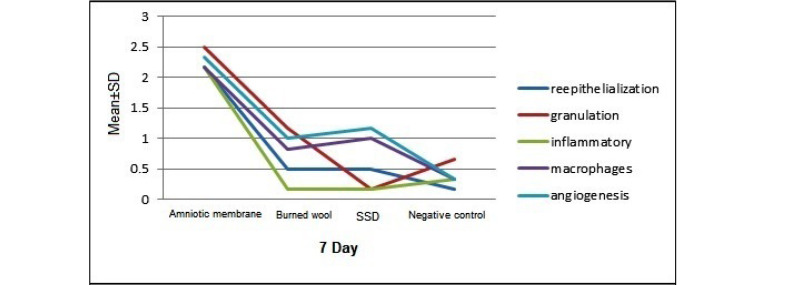
Scores of histopathological changes in different groups treated for second degree deep burn induced in the skin of male rats on days 7. **SSD:** Silver sulfadiazine ointment.

 On day 7, there was a significant difference between the human AM group compared with other groups in terms of re-epithelialization, granulation, inflammation, macrophages, and angiogenesis (P<0.05). The human AM was more effective in improving wound healing. The burned wool group showed significant improvement regarding angiogenesis (P<0.05) compared to other groups. There was a significant difference between the negative control group and SSD group in terms of angiogenesis and macrophage cell (P<0.05). Thus, the effectiveness of SSD ointment was better than the control group. There was a significant difference between the burned wool group and SSD group concerning granulation tissue formation (P<0.05), but there was no significant difference in other pathologic variables (Figure-1A). On day 14, human AM group was significantly different from the control groups (negative and positive) and burned wool in terms of re-epithelialization, granulation, inflammatory, macrophages, and angiogenesis (P<0.05). Therefore, the AM was more effective in improving burn wound healing than other groups. There was a significant difference between the burned wool and negative control groups regarding re-epithelialization and granulation (P<0.05). There was no significant difference between the SDD ointment and negative control (P>0.05).

**Figure 2:**
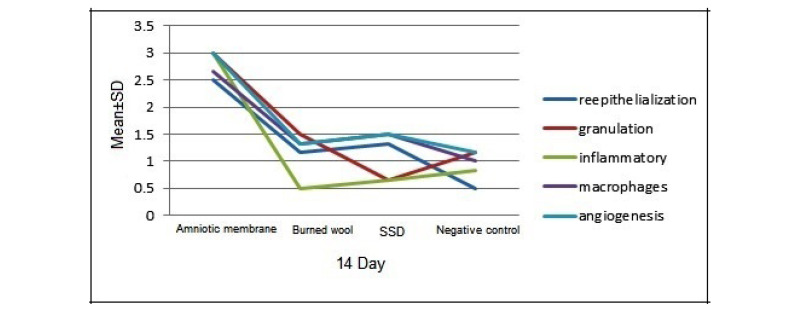
Scores of histopathological changes including reepithelialization, granulation, inflammatory, macrophages and angiogenesis between different groups treated the second degree deep burn induced in the skin of male rats on days 14. SSD: Silver sulfadiazine ointment.

**Figure 3:**
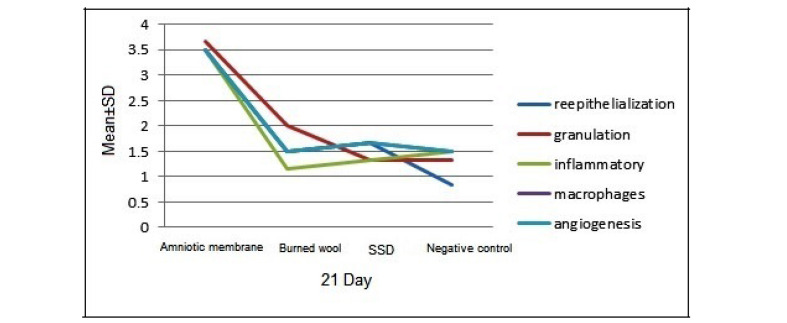
Scores of histopathological changes including reepithelialization, granulation, inflammatory, macrophages and angiogenesis between different groups treated the second degree deep burn in the skin of male rats on days 21.

**Figure 4:**
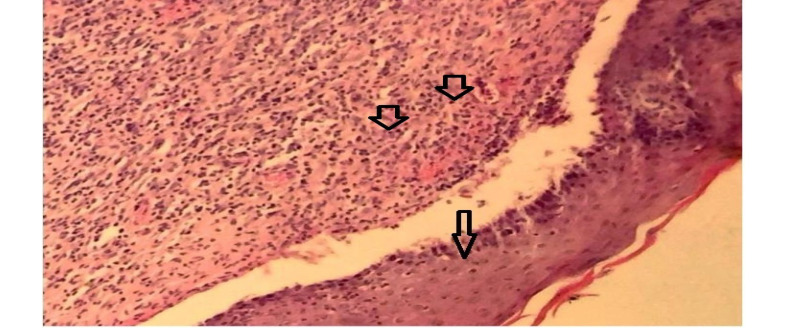
Wound burn section in the skin of male rat on day 21 treated with amniotic membrane showed completed reepithelialization (narrow arrow) with inflammatory cells (thick arrow). (H&E, ×200).

 A significant difference was found between the groups of burned wool and SSD ointment in the granulation formation (P<0.05), but there was no significant difference in other pathologic variables. Therefore, burned wool was more effective than SDD ointment in improving burn wound healing (Figure-1B). On day 21, there was a significant difference between the AM and other groups in re-epithelialization, granulation, inflammatory, macrophages, and angiogenesis (P<0.05, Figure-1C). AM showed better results in improving burn wound healing than the controls and burned wool group (Figure-2). SSD ointment was not effective in improving wound healing compared with the control group (P>0.05). Burned wool compared with SSD ointment showed better re-epithelization (P>0.05, Figures-3 and 4).

## Discussion

In the present study, human AM showed a significant therapeutic effect on deep second-degree burn compared to other groups. Pathological samples indicated an increase in re-epithelialization, granulation tissue, angiogenesis, and decreased inflammatory cells and macrophages on days 7, 14, and 21. AM is a natural scaffold with structural collagen extracellular matrix, biologically active cells and a wide variety of growth factors and cytokines as the main compounds involved in tissue regeneration, angiogenesis, and wound healing. Also, it produced tissue inhibitors of metalloproteinase and interleukins that are known to decrease inﬂammation [21]. Furthermore, human AM has other biological properties important for tissue restructure, including antimicrobial, anti-scarring, anti-fibrosis, and low immunogenicity [17]. Pessolato *et al*. concluded that using AM in second-degree burns in rats inhibited local inflammation and stimulated epithelial regeneration and production of collagen fibers [22]. The results of the previous studies mentioned above are consistent with our findings. A previous study was performed to evaluate the qualitative difference between AM and SSD ointment [20]. It was shown that the therapeutic effect of AM in the second-degree burn was better than SSD ointment [20]. They found that the rate of epithelialization was more rapid, and the duration of hospitalization, pain intensity, and frequency of dressing change were less [23]. Our study showed that the burned wool group compared with the control group increased angiogenesis in the wound site on day 7. Also, burned wool improved granulation formation and epithelialization on day 14 and epithelialization on day 21, which represents a significant effect on the improvement of second-degree burn. This effect can be due to the protein structure of wool, such as keratins, KAPs, and carbon composition that contribute to the recovery processes and increase wound repairing. We found significant improvement in granulation tissue formation in the burned wool group when compared with SSD group on days 7 and 14. This observation suggests that burned wool was more effective in wound healing than SSD ointment. The main structure of wool is keratin, and its most important amino acid is cysteine. The decomposition of wool produced five essential elements, including carbon, hydrogen, oxygen, nitrogen, and sulfur [12]. Thus, the present of proteins in wool leads to wound healing acceleration, such as egg yolk. Also, egg yolk reduces the size of burn wounds in the rabbit and rat model due to abundant proteins [24,25]. Hydrogels are widely used as debriding agents in the management of a variety of wounds. The main composition of the hydrogel is carbon and hydrogen [26]. Regarding the composition of the hydrogel and its similarity to the wool structure, it may be concluded that the effect of wool on the wound is due to its carbon and hydrogen. SSD ointment, compared to the control group, significantly increased the granulation tissue and reduced the macrophages on day 7, but there was no significant difference on days 14 and 21. In line with the current result, previous histological study revealed that SSD controlled the inflammation; however, it impaired the epithelialization and reduced the mechanical strength of the dermal tissue [27]. The reason for the lack of an increase in epithelialization in studies such as ours can be due to the toxic effect of silver in SSD ointment.

## Conclusion

 This study demonstrated that the application of human AM and burned wool could relieve the deep second-degree burns induced by the hot plate in the skin of rats. Histopathological evaluations indicated a significant increase in re-epithelialization, granulation tissue, angiogenesis, and reduced inflammatory and macrophages cells.

## Conflict of Interest

 The authors declare that they have no conflicts of interest.

**Figure 5:**
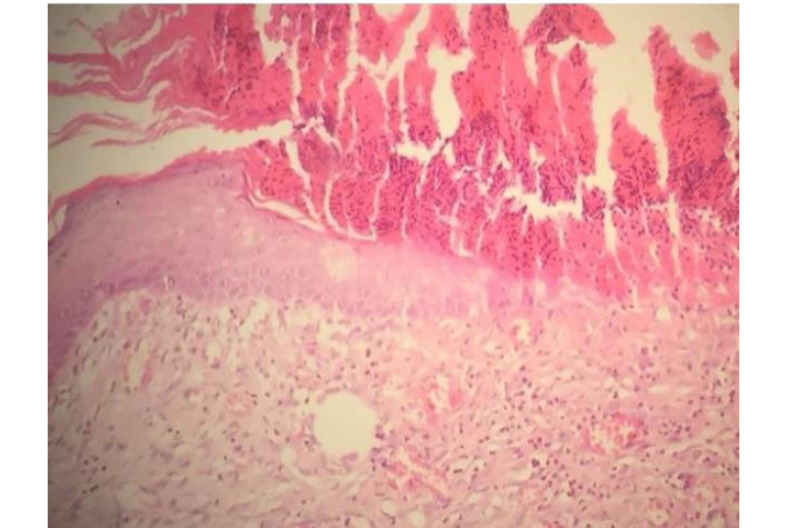
Wound burn section in the skin of male rat on day 21 treated with wool burned showed epithelization. (H&E, ×200).

**Figure 6:**
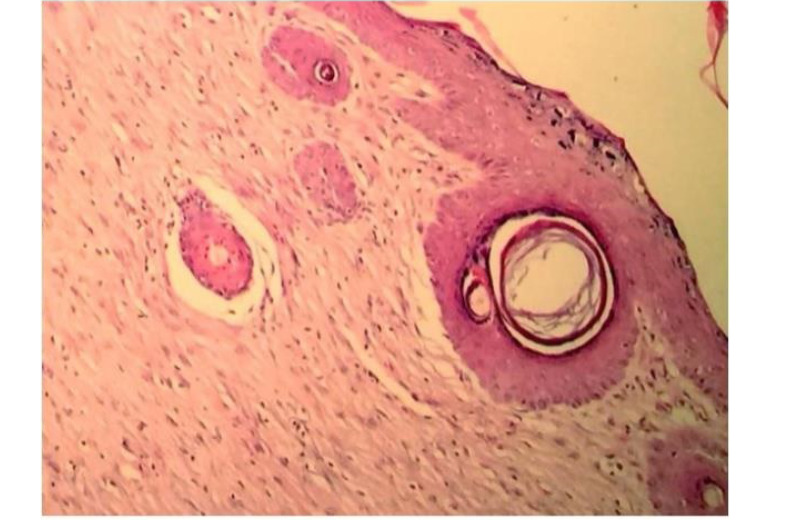
Wound burn section in the skin of male rat on day 21 treated with silver sulfadiazine showed epithelization with increasing collagen fibers. (H&E, ×200).
